# Systematic review of measurement properties of patient-reported outcome measures in patients with elbow-related orthopedic conditions

**DOI:** 10.1016/j.xrrt.2025.04.005

**Published:** 2025-05-06

**Authors:** Waleed Albishi, Hisham Alsanawi, Muhannad Alsharidah, Mohammed Alhuqbani, Zyad Aldosari, Omar Aldosari, Amr Elmaraghy

**Affiliations:** a Department of Orthopedic Surgery, College of Medicine, King Saud University, Riyadh, Saudi Arabia; b Department of Orthopedic Surgery, St Joseph's Health Centre, University of Toronto, Toronto, ON, Canada

**Keywords:** Elbow, Orthopedic surgery, Patient-reported outcome measure, PROMs, COSMIN, Validity

## Abstract

**Background:**

Elbow-related issues are common among adults and can significantly impact their daily activities and quality of life. Patient-reported outcome measures (PROMs) are increasingly being used nowadays to assess the subjective experience of patients with elbow problems. Identifying reliable, valid, and responsive tools is deemed necessary to be embedded in the assessment and treatment planning. This study aimed to identify currently available PROMs designed for people with elbow pathology and appraise, compare, and synthesize psychometric data supporting these PROMs.

**Methods:**

This review adhered to the methodology outlined by Cochrane and followed the principles set forth by the Preferred Reporting Items for Systematic Reviews and Meta-Analyses. Systematic searches were conducted in databases such as MEDLINE and Embase to identify studies related to PROMs for elbow diseases. The search was limited to papers published until July 2023. The inclusion criteria were specifically targeted toward adult populations, English-language publications, and research that provided information on the psychometric features of PROMs. The COSMIN Risk of Bias checklist was used to do data extraction and quality assessment.

**Results:**

Of the 6741 articles, 58 full-text articles were evaluated to determine their eligibility. Nineteen studies fulfilled the requirements for inclusion, providing information on 10 PROMs for elbow problems. PROMs such as the Disabilities of the Arm, Shoulder, and Hand, Quick Disabilities of Arm, Shoulder, and Hand, and Oxford Elbow Score have shown strong reliability and construct validity. Most PROMs did not have thorough qualitative evaluations and concept elicitation. The Patient-Rated Ulnar Nerve Evaluation and Patient-Rated Elbow Evaluation showed strong reliability and responsiveness, but the Elbow Self-Assessment Score and Kerlan-Jobe Orthopaedic Clinic score exhibited satisfactory reliability but lacked comprehensive qualitative assessment.

**Conclusions:**

Ten elbow PROMs were reviewed, showing psychometric diversity. Some PROMs are reliable and valid; others require further development and validation. Future studies could enhance qualitative assessments and standardization of PROM evaluation guidelines. A core outcome set for elbow-related PROMs could improve consistency and comparability in clinical research.

Health care providers encounter various elbow-related musculoskeletal problems in several clinical settings. Participation restrictions and activity constraints are key domains for computing, according to the World Health Organization's International Classification of Functioning, Health, and Disability.^21^ Elbow conditions encompass the surrounding tendons, muscles, ligaments, and neural tissues.^14^ The 3 most essential factors determining function are muscle strength, range of motion, and pain when performing tasks. Problems with these qualities can cause functional loss that can hinder daily tasks and lead to disability. Hence, functional loss may be a major problem in itself or may have an adverse effect on quality of life.^30^

Patient-reported outcome measures (PROMs) are used more frequently to assess outcomes in patients with elbow pathology as the number of patient-centered health care models continues to increase.^15^^,^^25^ PROMs present a patient's subjective experience of their health condition and are essential for evaluating functional outcomes and quality of life.^1^^,^^11^ Various PROMs have been used in orthopedic research, and determining the most suitable PROM for a certain population and condition can be challenging. Ideally, the PROM should be reliable, valid, and responsive to changes in clinical status while not being difficult to complete.^10^^,^^25^ Numerous methods are used to assess the quality of psychometric evidence for PROMs.^19^^,^^27^ For instance, the COSMIN checklist is a widely used appraisal tool for evaluating the methodological quality of research and examining the psychometric features of instruments for measuring health-related quality of life.^19^^,^^27^^,^^28^ Terwee et al^32^ established criteria for evaluating the quality of PROM's psychometric evidence in 2010. These criteria have been since applied to assess different PROMs.^5^^,^^9^^,^^15^^,^^26^^,^^36^^,^^37^

This study aimed to identify currently available PROMs designed for people with elbow pathology and appraise, compare, and synthesize psychometric data supporting these PROMs.

## Methods

### Literature search strategy

The present systematic review was formulated using the rigorous Cochrane review methodologies while adhering to the Preferred Reporting Items for Systematic Reviews and Meta-Analyses guidelines. The existing literature from various databases from their inception until July 2023 was systematically reviewed to present all available PROMs for assessing orthopedic pathologies in and around the elbow. The MEDLINE and Embase databases were subsequently searched. To facilitate the research process, terms and keywords encompassing the elbow and all psychometric attributes examined in the paper published by Terwee et al were used. This review includes an examination of the search outcomes and published studies without any temporal constraints.

### Eligibility criteria

The inclusion criteria for research articles focused on studies using PROM for elbow diseases, defined as a variety of musculoskeletal conditions affecting the elbow joint and its surrounding structures, including tendons, muscles, ligaments, and neural tissues; publications without any timeframe restrictions; publications in the English language; availability of full-text articles; reporting of either principle development, concurrent revalidation, or prospective cohorts; inclusion of an adult population aged ≥18 years; reporting of psychometric properties of PROMs; and utilization of PROMs in clinical or research settings. The exclusion criteria were as follows: studies published in languages other than English and studies that used incorrect research methodologies or if they did not report on 1 or more psychometric measurement properties of PROMs (eg, internal consistency, reliability, validity, responsiveness, or content validity) as defined by the COSMIN criteria. Systematic reviews, meta-analyses, economic analyses, animal experiments, cadaver studies, narrative reviews, and editorial pieces were also excluded from the analysis. The initial screening of abstracts for the included papers was conducted using the Rayyan search engine.

### Search screening

**Figure 1:**
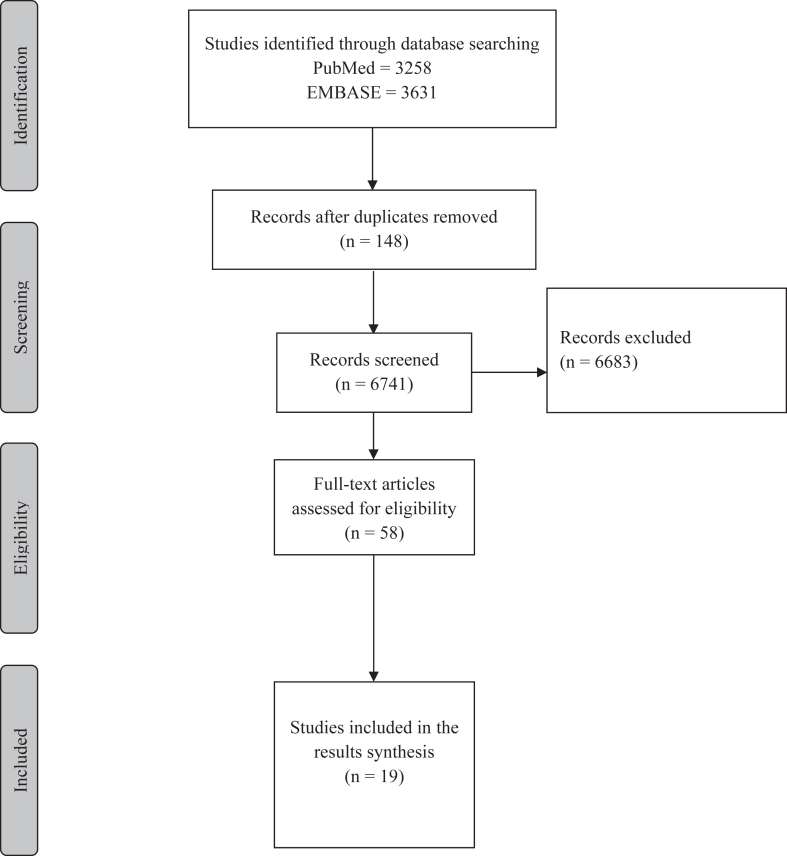
Preferred Reporting Items for Systematic Reviews and Meta-Analyses flowchart of article selection through different screening and eligibility phases for inclusion in a systematic review.

The detected records were exported to the EndNote X9 reference management software for further analysis. Duplicate records were eliminated. Three independent reviewers evaluated the titles and abstracts for eligibility using the Rayyan search engine. The reviewers obtained full texts of potentially eligible articles, and 2 authors blindly/independently reviewed all abstracts according to aforementioned criteria. To address any discrepancies between the 2 reviewers, a third evaluator independently examined the selected articles. Subsequently, the complete texts of the studies were comprehensively examined to ensure adherence to the predetermined inclusion and exclusion criteria before data extraction. Additional publications were selected from the reference lists of included studies. Disagreements were resolved by establishing a consensus or engaging a fourth reviewer when necessary (Figure 1).

### Data extraction

Data, including title, author, publication year, country, study design, PROM tool, language, and condition, were extracted. In addition, demographic information, such as sample size and mean age or age range, was collected. The psychometric data of the measurement properties examined in the included studies were evaluated based on the quality standards set by Terwee et al.^7^^,^^27^ The rating scale encompassed various quality characteristics pertaining to internal consistency, reliability, measurement errors, content validity, criterion validity, construct validity (convergent and divergent), and responsiveness. Properties in the literature with missing information were rated by placing a hyphen (−).^24^

### Quality and bias assessments

The COSMIN Risk of Bias checklist was used to analyze the study's level of methodological rigor.^18^ Each of these steps ensured a rigorous and comprehensive approach to conducting this systematic review, providing robust results and conclusions on PROMs in upper extremity orthopedics. The primary objective of assessing quality was to evaluate the potential presence of bias in the studies included in the systematic review. The concept of “risk of bias” aligns with the Cochrane methodology for performing systematic reviews of trials and diagnostic investigations. This term refers to the credibility of a study's findings based on the quality of its methodology.

The assessment of numerous measurement parameters was the primary focus of evaluating the methodological quality of the articles. These features included internal consistency, reliability, measurement error, content validity, construct validity, criterion validity, and responsiveness. Other properties included construct, criterion, and construct validities. The latest version of the COSMIN guidelines and the risk-of-bias checklist published in 2018 and 2017, respectively, were used in this study. This grading method incorporating 5 distinct options for responses (very good, adequate, doubtful, inadequate, and not applicable) was used to assess the different properties of various tools in 10 different aspects. The overall evaluation of the methodological rigor of each study was established for each property by selecting the lowest grade among the individual components of that property. For instance, if a certain property such as content validity earned a very good rating on 1 item but an inadequate rating on another, the overall methodological quality relevant to that property would be considered inadequate. Using the updated COSMIN grading approach, 2 reviewers separately assessed the publications to determine their level of methodological excellence. In cases of disagreement between the 2 raters, a consensus was reached. In situations where an agreement could not be reached through discussion alone, the participation of a third person who was not involved in either side of the dispute was sought to facilitate resolution.

### Statistical analysis

Statistical analyses were not performed. The absence of a meta-analysis can be attributed to the overall variability observed in the data.

## Results

### Study selection

In total, 6741 unique articles were screened; 58 full-text articles of which were assessed for eligibility. Nineteen studies reporting on elbow PROMs were included in the scoping review. Thirteen studies describing the measurement properties of 10 PROMs met the inclusion criteria for PROM validation.^1^, ^2^, ^3^^,^^6^^,^^13^^,^^15^, ^16^, ^17^^,^^20^^,^^22^^,^^23^^,^^33^^,^^34^

### Characteristics of studies reporting PROM outcomes

Table I presents the selection of PROMs that are specifically used for assessing the elbow joint. The Kerlan-Jobe Orthopaedic Clinic shoulder and elbow score consists of 10 items measuring functional status, with responses on a 0-10 scale, and is self-administered. The Patient-Rated Tennis Elbow Evaluation assesses pain and functional status through 15 items and 2 subscales, also on a 0-10 scale, and is self-administered. The self-rated American Shoulder and Elbow Surgeons score covers activities of daily living and pain over 17 items with 2 subscales. Similarly, the Patient-Rated Elbow Evaluation (PREE) has 20 items with 2 subscales, measuring pain and function on a 0-10 scale, and is self-administered.

**Table I:** Patient-reported outcome measure–specific information intended for use on the elbow joint.

Name of measurement	Domains measured	No. of items and subscales	Response options	Administration mode	Target disease
Kerlan-Jobe Orthopaedic Clinic shoulder and elbow score^2^	Functional status	10 items	Scale of 0-10	Self-administration	Upper extremity functional status among athletes
Patient-rated tennis elbow evaluation^19^	Pain, functional status	15 items, 2 subscales	Scale of 0-10	Self-administration	Lateral epicondylitis
The American Shoulder and Elbow Surgeons score^22^	Activity of daily living, pain	17 items, 2 subscales		Self-administration	Shoulder and elbow condition regardless of pathology
Patient-rated elbow evaluation^20^	Pain, function	20 items, 2 subscales	Scale of 0-10	Self-administration	Elbow complaints
The Elbow Self-Assessment Score^6^	Pain, function, quality	22 items, 3 subscales	Scale of 0-10, + clinical	Self-administration	Elbow disorders
Oxford Elbow Score^11^^,^^19^	Pain, function, sociopsychology	12 items, 3 subscales (elbow function, elbow pain, and a social–psychological domain)	5 response options	Self-administration	Elbow disorders
Patient-rated ulnar nerve evaluation^21^	Pain, symptoms, activity	19 items	Scale of 0-10	Self-administration	Ulnar nerve compression at the elbow
Ulnar neuropathy at the elbow^25^	Symptoms	9 items	5 options	Self-administration	Ulnar neuropathy at the elbow
Disabilities of Arm, Shoulder and Hand^15^	Daily activities, symptoms, social function, work function, sleep, and confidence	30 item, 7 subscales	Items are scored from 1 to 5 (where 5 represents the greatest severity)	Self-administration	Upper extremity disorders
Quick Disabilities of Arm, Shoulder and Hand^5^	Upper extremity, activity participation, activities of daily living, sensory	11 items, 6 subscales	Items are scored from 1 to 5 (where 5 represents the greatest severity)	Self-administration	Upper extremity disorders

The Elbow Self-Assessment Score is a self-administered tool with 22 items across 3 subscales, addressing pain, function, and quality, with a scale of 0-10 plus clinical input. The Oxford Elbow Score evaluates pain, function, and sociopsychological aspects using 12 items and 3 subscales and provides 5 response options for self-assessment. The Patient-Rated Ulnar Nerve Evaluation is designed for ulnar neuropathy at the elbow (UNE) and comprises 19 items focused on pain, symptoms, and activity level rated on a 0-10 scale for self-administration. More specifically, the self-rated Disabilities of the Arm, Shoulder, and Hand (DASH) scale includes 30 items over 7 subscales, capturing daily activities, symptoms, social function, work function, sleep, and confidence, scored from 1 to 5. Finally, self-administered Quick Disabilities of Arm, Shoulder, and Hand (QuickDASH) is a shortened version of the DASH and comprises 11 items with 6 subscales, assessing upper extremity activity, participation, activities of daily living, and sensory aspects, scored from 1 to 5.

### Quality of PROM development studies

As presented in Tables II and III, this study evaluated the quality of development of various PROMs for elbow conditions, guided by criteria including clarity of the construct, origin of the construct, target population specificity, context of use, sample representation, and performance of cognitive interview (CI) studies.

**Table II:** Quality of patient-reported outcome measure (PROM) development for the elbow.

PROM	Language in which the PROM was developed	PROM design							Cognitive interview (CI) study2			Total PROM development
		General design requirements					Concept elicitation1	Total PROM design	General design requirements	Comprehensibility	Total CI study	
		Clear construct	Clear origin of construct	Clear target population for which the PROM was developed	Clear context of use	PROM developed in sample representing the target population			CI study performed in sample representing the target population			
Disabilities of Arm, Shoulder and Hand^38^	English	V	V	V	V	V	I	I	Yes	A	I	I
Quick Disabilities of Arm, Shoulder and Hand^3^	English	V	V	V	V	V	I	I	Yes	A	I	I
Kerlan-Jobe Orthopaedic Clinic shoulder and elbow score^28^	English	V	V	V	V	V	I	I	Yes	I	I	I
Patient-rated tennis elbow evaluation^13^	English	V	V	V	V	V	I	I	No	–	–	I
The American Shoulder and Elbow Surgeons score^38^		V	V	V/I	V	V	I	I	No	–	–	I
Patient-Rated Elbow Evaluation^20^	English	V	V	V	V	V	I	I	No	–	–	I
The Elbow Self-Assessment Score^6^	English	V	V	V	V	V	I	I	No	–	–	I
Oxford Elbow Score^17^	English	V	V	V	V	V	I	I	Yes	V	I	I
Ulnar neuropathy at the elbow^25^	Clinical	V	V	V	V	V	I	I	No			I
Patient-rated ulnar nerve evaluation^21^	English	V	V	V	V	V	I	I	Yes	V	I	I

*V*, very good; *I*, inadequte; *(−)*, missing information; *PROM*, patient-reported outcome measure.

**Table III:** Quality of studies on measurement properties for PROMs for the elbow joint.

PROM	Content validity					Structural validity	Internal consistency	Reliability	Measurement error	Criterion validity	Construct validity		Responsiveness		
	Asking patients			Asking experts											
	Relevance	Comprehensiveness	Comprehensibility	Relevance	Comprehensiveness						Convergent validity	Known groups validity	Comparison with gold standard	Comparison with other instruments	Comparison before and after intervention
Disabilities of Arm, Shoulder, and Hand^20^^,^^38^	D	D	D	D	D	A	V	V		V	V		V	V	V
Quick Disabilities of Arm, Shoulder, and Hand^3^	D	D	D	D	D	V	V	A	A	V	V	V	V	V	V
Kerlan-Jobe Orthopaedic Clinic shoulder and elbow score^28^	I	I	I	D	D	V		V	A		V			V	V
Patient-Rated Tennis Elbow Evaluation^13^^,^^19^						V	V	V	V		V			V	-
The American Shoulder and Elbow Surgeons score^38^	I	I	I	D	D	V	V	V	V		V	V		V	
Patient-Rated Elbow Evaluation^39^	I	I	I	A	A	A	V	V		V	V			V	
The Elbow Self-Assessment Score^6^	I	I	I	D	D		V	V			V			V	
Oxford Elbow Score^17^	D	D	D	D	D	V	V	V	V	V	V			V	
Ulnar neuropathy at the elbow^26^	I	I	I	D	D	V	V	V			V	V		V	V
Patient-Rated Ulnar Nerve Evaluation^21^	A	A	A	D	D	A		V			V	V		V	

*PROM*, patient-reported outcome measure; *V*, very good; *A*, adequate; *D*, doubtful; *I*, inadequate; *(−)*, missing information.

The DASH instrument was developed in English with very good clarity of construct and origin. It is also specific to the intended population and is constructed in a relevant context using an adequate sample. The PROM's overall design was rated as inadequate despite a CI study performed with the sample representing the target population, indicating room for improvement. The QuickDASH scored very well on the same criteria, with a development process that included an adequate CI study. However, the overall design was deemed inadequate, suggesting the need for enhanced development procedures.

The Kerlan-Jobe Orthopaedic Clinic shoulder and elbow score, American Shoulder and Elbow Surgeons score, and PREE displayed a consistent quality of development with very good ratings across the board; however, their CI studies were deemed inadequate, indicating a significant shortfall in qualitative assessment. Although the Elbow Self-Assessment Score demonstrated very good clarity in construction and development, it was not supported by a CI study, rendering its comprehensibility inadequate. The Oxford Elbow Score was an exception, showing a very good development process across all domains, including a very good CI study, highlighting a comprehensive approach to its creation. For UNE and Patient-Rated Ulnar Nerve Evaluation, the development criteria were very good, and the absence of a CI study indicates an inadequate understanding of the qualitative efficacy of the PROM.

### Psychometric properties and adequacies of measurement properties

As displayed in Table IV, the Patient-Rated Ulnar Nerve Evaluation demonstrated high reliability with intraclass correlation coefficients (ICCs) exceeding 0.90 for all subscales, except for usual activities, which was 0.87. The total score reached an ICC of 0.98, indicating excellent test–retest reliability. The measurement errors, represented by the standardized response mean and minimal detectable change (MDC), were 3.1 and 7.2% of the total score, respectively. The evaluation can differentiate between clinically meaningful subgroups, with all subscale sensitivities to change scores of <0.90 but with a notable total score change sensitivity standardized response mean [SRM] of 1.55.

**Table IV:** Psychometric properties and adequacies of measurement properties.

Name of measurement	Reliability	Internal consistency	Structural validity	Measurement error	Construct validity	Responsiveness	Sensitivity to change
Patient-Rated Ulnar Nerve Evaluation^21^	ICC for total score = 0.98 ICC for all subclasses >0.9 (except usual activities = 0.87)	–	–	SEM and MDC were 3.1 and 7.2, respectively, for the total score	It was able to discriminate between clinically meaningful subgroups determined by an independent evaluation assessing work status, residual symptoms, motor recovery, sensory recovery, and global improvement, *P* < .01.	All subscales (all SRM <0.90) and the total score (SRM = 1.55).	–
Ulnar neuropathy at the elbow^25^	*r* = 0.97 for total score	0.83 (0.87 w/o CTS)	–	–	*r* = 0.32 (0.35) with electrophysiological severity scale, *r* = 0.52 (0.65) with clinical severity scale	Moderate to large (0.5-0.8) 0.46	–
Kerlan-Jobe Orthopaedic Clinic shoulder and elbow score^28^	ICC = 0.83 and 0.88	NA	*R*2 = 74%, 10 factors	MDC (MDC 95%) of 6.7.	*r* = 0.84 and 0.86	–	–
Patient-rated tennis elbow evaluation^19^	*r* = 0.87 for total score, 0.92 for pain subscale, 0.87 for function: special activities, 0.77 for function: usual activities	0.95	–	(MCID) = 11, (MDC) = 9	*r* = 0.84 between total score and Thomsen test and r = 0.75 with DASH	*r* = 0.84 between total score and Thomsen test and *r* = 0.66 with DASH, *r* = 0.84 between special activities score and Thomsen test and *r* = 0.92 with DASH	–
The American Shoulder and Elbow Surgeons score^38^	ICC: 0.84	0.93	*R*2 = 74.4%, 4 factors	MDC 9.7	(*r* = 0.72-0.91) with the PREE total score, the PREE pain score, the PREE function score, the pASES-e pain score, and the DASH total score	(*r* > 0.70)	SRM (pASES-e pain scale, 1.2; 95% CI: 1.0, 1.4 and pASES-e function scale, 1.1; 95% CI: 0.9, 1.3)
Patient-rated elbow evaluation^19^	ICC: 0.6-0.88, >0.9 for sum score	0.95	*R*2 = 77.2%, 4 factors	–	(*r* = 0.72-0.91) with the PREE total score, the PREE pain score, the PREE function score, the pASES-e pain score, and the DASH total score	(*r* > 0.70)	SRM 1.6; 95% CI: 1.4, 1.8
The Elbow Self-Assessment Score^6^	ICC between 0.71 and 0.81 for all subscales	>0.83	–	–	*r* = at least −0.80	The SCC was 0.73 for pain, 0.84 for function, and 0.72 for elbow-related quality of life.	–
Oxford Elbow Score^17^	ICC for total score was 0.96.	0.83 for elbow function, 0.91 for pain, and 0.90 for social–psychological domains		MCIDs for the OES function scale and the DASH were similar (around 10) but were larger for the OES Pain and Social–Psychological scales (around 18), reflecting their lower (ie, poorer) baseline scores and larger effect sizes. However, the MCIDs were only consistently larger than the MDCs for the OES pain domain.	*r* = −0.91 between OES total and QuickDASH scores, 0.76 between OES total scores and Single Assessment Numeric Evaluation-Function values	*r* = −0.85 (responsiveness for improvement) and *r* = −0.88 for (responsiveness for deterioration)	–
Disabilities of Arm, Shoulder, and Hand^38^	ICC: 0.93, >0.9 for sum score	0.97	*R*^2^ = 71.3%, 5 factors	–	(*r* = 0.72-0.91) with the PREE total score, the PREE pain score, the PREE function score, the pASES-e pain score, and the DASH total score	Moderate correlations (*r* = 0.41-0.62) were observed between the DASH change scores and the change scores of PREE and SF36.	SRM (1.6; 95% CI: 1.5, 1.8)
Quick Disabilities of Arm, Shoulder, and Hand^3^	0.7 for symptoms, 0.92 for function, 0.91 for total score	0.92	–	–	*r* = 0.91 between OES total and QuickDASH scores	*r* = −0.85	–

*ICC*, intraclass correlation coefficient; *SEM*, standard error of measurement; *MCID*, minimal clinically important difference; *MDC*, minimal detectable change; *DASH*, Disabilities of the Arm, Shoulder, and Hand; *PREE*, Patient-Rated Elbow Evaluation; *OES*, oxford elbow score; *CI*, confidence interval; *pASES-e*, Patient-Reported American Shoulder and Elbow Surgeons Elbow Questionnaire; *SCC*, Spearman Correlation Coefficient.

UNE showed an impressive *r* value of 0.97 for total score reliability and moderate-to-large construct validity, as indicated by correlations with electrophysiological and clinical severity scales. However, responsiveness was moderate, with an SRM of 0.46. The Kerlan-Jobe Orthopaedic Clinic shoulder and elbow scores had good reliability, with ICCs of 0.83 and 0.88, respectively, and the MDC was 6.7. Construct validity correlations with other clinical measures were good, with *r* = 0.84 and *r* = 0.86, respectively, although responsiveness data were not provided.

The Patient-Rated Tennis Elbow Evaluation showed good reliability for the total score (*r* = 0.87), particularly for the pain subscale (*r* = 0.92). The construct validity of the tool was confirmed by strong correlations between the total score and other clinical tests. However, responsiveness information was unavailable. For the American Shoulder and Elbow Surgeons score and PREE, the ICCs were very good, ranging from 0.6 to 0.9 for sum scores, indicating high reliability. Construct validity was also established, with a very good correlation with other assessments, although the responsiveness data were not detailed.

The ICCs for the Elbow Self-Assessment Score ranged from 0.71 to 0.81 across subscales, with overall good reliability and construct validity compared with other scales. Responsiveness was good, with an SRM of 1.6. The total ICC of the Oxford Elbow Score was 0.96. Construct validity was strong, with high correlations with various clinical measures, and responsiveness was excellent with SRMs for pain and function scales of >1.0. The ICC for the DASH was 0.93, indicating excellent reliability. QuickDASH also demonstrated very good reliability, with ICCs ranging from 0.7 to 0.92. Construct validity was confirmed through moderate correlations with changes in other validated scores.

## Discussion

Our systematic review identified 10 PROMs in patients with elbow conditions/diseases. These tools differ widely in terms of the domains and conditions being evaluated (Table I). This study evaluated the quality of PROM development, quality of content validity, and risk of bias according to the COSMIN checklist. Herein, psychometric properties related to elbow usage as reported in the literature are presented.^18^

When assessing the quality of development, all PROMs were rated as inadequate in terms of concept elicitation. This was because most studies did not provide a reason for not conducting interviews and focus groups in which video or audio recordings were used to fully capture their content and context. However, most studies were rated as very good upon assessment of the general developmental study design. Cognitive interviews play an important part in the development and validation of PROMs, as highlighted by the COSMIN checklist. The PROM's instructions, items, response options, and recall periods must be clearly understood by the target population to prevent misinterpretation or confusion that could compromise data quality. Cognitive interviews improve the comprehensibility, reliability, and validity of PROMs by identifying and addressing ambiguities or cultural nuances, thereby enhancing their effectiveness for intended purposes.

Ensuring that stakeholders are approached when developing the relevance, comprehensiveness, and comprehensibility of a PROM among patients and professionals is crucial (Table III). Most PROMs demonstrated inadequate or doubtful ratings in the content validity quality evaluation. Only the PREE was rated with adequate relevance and comprehensiveness in the professionals' section on quality of content validity. Furthermore, the Patient-Rated Ulnar Nerve Evaluation has secured from patients’ adequate relevance and comprehensiveness ratings (Tables II and IV).

### Strengths and limitations

This study has several strengths. To the best of our knowledge, this is the first study to integrate data to provide an overall evaluation of PROMs and systematically assess the methodological quality and psychometric evidence of PROMs in patients with elbow problems. In addition, the study used the COSMIN checklist, which has been reported as effective and widely used in previous studies.^5^^,^^8^^,^^9^^,^^12^^,^^31^^,^^36^^,^^37^

However, this study has possible limitations. First, we did not evaluate the study findings using performance-based or clinician-reported outcome measures. Second, the literature was frequently inadequately reported, which complicated our evaluations, if not rendered them impossible in certain instances. This impaired our ability to precisely assess these instruments. As previously mentioned, the instruments under review demonstrated significant variation regarding both the quantity of items and the nature of the domains being evaluated. Thus, although the DASH provides the strongest evidence of its properties, individuals should be consulted (Table IV) to ascertain which instrument and its associated domains suit their requirements the most. The instruments that provide the strongest psychometric evidence may not always be suitable for a particular research inquiry or patient population. However, caution should be exercised when considering PROMs with negative evidence, indicating the absence of a particular property.

This study differentiates itself from prior research by encompassing a wider scope and offering a thorough review of 10 PROMs for multiple elbow-related conditions. In contrast to the study by Bertram et al,^29^ which reported 3 patient-administered rating scales. In addition, while Beor et al^4^ focused on creating a disease-specific tool for rheumatoid arthritis patients and conducted comparisons with established scales (eg, HSS and Mayo Clinic), our research addresses a broader spectrum of elbow pathologies. It offers insights into current PROMs and highlights developmental gaps, including the necessity for CIs and qualitative assessments.

Our study extends beyond the Zarezadeh et al^38^ study on distal humerus fractures, which assessed outcome measures such as Mayo Elbow Performance Score and DASH in a fracture-specific context. Their study identified inconsistencies in reporting and advocated for the use of multiple outcome measures for fractures. In contrast, our research adopts a comprehensive approach by examining PROMs for fractures, tendinopathies, and arthritis. Adhering to Terwee and COSMIN guidelines allows for the identification of areas needing improvement in PROM development.

### Future research

The first attempt to standardize the PROM assessment procedure was the consensus-based COSMIN checklist.^18^ Comparing the PROMs among the investigations was possible based on such guidelines. Thus, future research should use the COSMIN checklist.

The evidence was synthesized in the present study by combining Terwee's psychometric criteria and the COSMIN methodological quality criteria. Despite being widely used, these 2 criteria have not been jointly developed, which accounts for some of their disparities. Terwee's guidelines address only overall hypothesis validity, whereas the COSMIN checklist considers 3 dimensions of hypothesis validity as follows: convergent, discriminant, and discriminative. We support the development of methodological guidelines for conducting thorough analyses of psychometric qualities of PROMs. High-quality research on the development and assessment of PROMs should be conducted in the future.

Further research is needed to analyze and synthesize all research and determine the conditions needed to make evidence-based recommendations. The results of this study may also depend on patient variables.^32^ One instrument was unlikely to respond well when the patient's condition improved slightly after treatment. Increasing patient variation makes cross-study comparisons problematic; therefore, more stringent procedures are required to eliminate subjective bias. Moreover, caution should be exercised when analyzing reliability. Time intervals should be sufficiently long to prevent bias yet sufficiently short to guarantee that patients have not changed the construct to be tested. The construct used to measure and target a population determines the time interval. In cases of rapid illness progression or in elderly people with impaired memory, a 2-week period may be insufficient.

In addition, constructing a core outcome set (COS) specifically for PROMs used in individuals with elbow conditions is possible. A COS or core outcome collection is a predetermined collection of outcomes or measures that are agreed upon and used to conduct and report clinical research in specific clinical studies.^35^ A COS enables clinical researchers analyzing PROMs to make meaningful comparisons between studies addressing comparable issues. In addition, it helps prevent inefficient and unreliable findings by using outcome measures in clinical research that have well-established and recognized qualities. Considering the diversity of PROMs used in patients with elbow disorders and their varying levels of quality, conducting research is necessary to establish a COS.

## Conclusion

Ten PROMs were evaluated for the assessment of elbow concerns. The psychometric characteristics and developmental quality assessment were examined and reported. Most PROMs exhibited insufficient qualitative assessments and concept elicitation. Despite these limitations, almost all PROMs demonstrated excellent psychometric characteristics. Future research may improve qualitative evaluations and the standardization of PROMs assessment procedures. A COS for elbow-related PROMs could enhance uniformity and comparability in clinical studies.

## Disclaimers:

Funding: No funding was disclosed by the authors.

Conflicts of interest: The authors, their immediate families, and any research foundation with which they are affiliated have not received any financial payments or other benefits from any commercial entity related to the subject of this article.
